# Autoplasty

**Published:** 1845-05

**Authors:** 


*Autoplasty.*—M. Blandin presented to the Academy of Medicine a woman from whom he had removed a cancer of the lower eyelid. She had been operated on three times by M. Paiault of Rennes. The cancer having relapsed a third time, M. Puiault sent the patient to M. Blandin. The disease involved the whole of the lower eyelid, the corresponding region of the upper part of the nose, and a part of of the upper eyelid. M. Blandin after having extirpated all the diseased parts borrowed a flap from the forehead, to replace, in part, the very considerable loss of substance caused by the operation, this, Mr. Blandin said, he did much less with the view of repairing the loss of substance, which was not completely accomplished, than in the hope of obtaining, by the transplantation of a sound structure on the affected parts, a modification of a structure and of nutrition which might lesson the chance of relapse. This was M. Blandin's chief object in practising autoplasty in this case, and he thought he had been so fortunate as to attain it, as the operation had been performed three months, all the parts were united, and no sign of a return of the disease existed. M. Blandin felt the greater confidence in anticipating a favorable issue to the operation, as he had effected a complete cure in a similar case, by the same method, on a man whom he had operated on eighteen months ago.—***Dublin Med. Press, from Gaz. Med.***

